# Seed Priming by Low-Dose Radiation Improves Growth of *Lactuca sativa* and *Valerianella locusta*

**DOI:** 10.3390/plants13020165

**Published:** 2024-01-08

**Authors:** Maria Cristina Sorrentino, Angelo Granata, Martina Cantalupo, Lorenzo Manti, Mariagabriella Pugliese, Simonetta Giordano, Fiore Capozzi, Valeria Spagnuolo

**Affiliations:** 1 Department of Biology, University of Naples Federico II, 80126 Naples, Italy; mcristinasorrentino@gmail.com (M.C.S.); angelo.granata@unina.it (A.G.); mart.cantalupo@studenti.unina.it (M.C.); giordano@unina.it (S.G.); valeria.spagnuolo@unina.it (V.S.); 2 Department of Physics, University of Naples Federico II, 80126 Naples, Italy; lorenzo.manti@unina.it (L.M.); mariagabriella.pugliese@unina.it (M.P.)

**Keywords:** ionizing radiations, lettuce species, antioxidants, mean germination time

## Abstract

Valerian salad and lettuce are edible species that are easy to grow rapidly, and have traits useful for commercial purposes. The consumption of these species is increasing worldwide for their nutritional properties. Seed germination and seedling development are critical stages in the life cycle of plants. Seed priming, including the use of high-energy radiation, is a set of techniques based on the idea that low stress levels stimulate plant responses, thereby improving seed germination and plant growth. In this study, we evaluated in hydroponic culture (i) the germination performance; (ii) morphological traits; and (iii) antioxidant and phenol contents at different endpoints in *Lactuca sativa* and *Valerianella locusta* that were developed from seeds exposed to X-rays (1 Gy and 10 Gy doses). Under radiation, biomass production increased in both species, especially in lettuce, where also a reduction in the mean germination time occurred. Radiation increased the level of phenols during the first growth weeks, under both doses for lettuce, and only 1 Gy was required for valerian salad. The species-specific responses observed in this research suggest that the use of radiations in seed priming needs to be customized to the species.

## 1. Introduction

Soilless agriculture is one of the main approaches to cope with depletion of soil fertility [1]. In fact, the widespread use of hydroponics, especially for edible species that have fast reproductive cycles like ready-to-eat salads, is becoming increasingly important, and represents a feasible opportunity to satisfy increasing market demand [2,3,4]. Moreover, especially for leafy vegetables, soilless systems can reduce soil contaminants and improve sanitary and nutritive qualities with respect to traditional soil culture [5].

Efficient, fast, and synchronized seed germination and seedling establishment are fundamental prerequisites for the success of any crop in agriculture [6,7]. Seed priming is a set of techniques that consist of seed invigorating procedures, which are employed before the initial phase of germination [6]; priming processes provide an innovative, cost-effective, and environmentally sound solution that is an alternative to chemical pesticides and fertilizers for improving seed quality and crop health, and also for attaining higher yields. Seed germination is a crucial process involving different metabolic events, which are traditionally grouped into three phases. The first phase, known as the imbibition phase, involves quick water uptake through forces driven by seeds. During this phase, the dormancy of the seed is broken, and many metabolic pathways restart, and DNA and mitochondria that were damaged under drought conditions are repaired. The second phase, in which water uptake occurs in a lower measure, is called the activation phase, since this period is highly active, physiologically and metabolically. This leads to maturation of mitochondria (with subsequent ATP synthesis), transcription and protein synthesis, and hydrolysis of stored macromolecules into molecules that are required for radicle emergence. The third phase is the germination phase, which is characterized by the rupture of the seed coat and primary root outgrowth [1,2,5]. Seed priming treatment, which is carried out before sowing seeds, determines the hydration level of seeds that must be abundant enough to enable metabolic events to take place before germination, while preventing the occurrence of radicle emergence [7]. Currently, different priming techniques are applied sensu stricto (i.e., based on controlled water absorption) to provide better seed quality, such as hydro-priming, halo-priming, osmo-priming, hormone or growth enhancer priming, micronutrient seed priming (i.e., nutripriming using organic or inorganic substances), and biopriming using microorganisms [4]. In a broader sense, seed priming techniques also include physical methodologies applied to dry seeds, which, compared to classical approaches based on controlled water absorption, offer the advantage of overcoming problems related to post-treatment storability of the seeds [8]. Specifically, different types of electromagnetic radiation, such as gamma rays [9], high-energy electrons [10], ultrasonic radiation [11], microwaves [12,13], and UV radiation [14]; high-energy radiations have been used as alternative seed priming treatments to mitigate microbial infestations, or for improving seed germination and plant growth [15,16,17].

It is known that small doses of ionizing radiation stimulate the growth of some plant species, probably due to reducing microorganism proliferation that can slow down the growth of seedlings, and at the same time activating responses to counteract stress. Such a stimulating effect was observed, for instance, in *Phaseolus vulgaris* (under X-rays at 10 Gy) and *Phoenix dactylifera* (X-rays at 1 Gy) [18,19,20]. In the present research, we tested low doses of X-rays as seed priming treatments in *Valerianella locusta* and *Lactuca sativa*, evaluating different end points: (i) the germination percentage and the mean germination time; (ii) the above-ground biomass, plant height, root length, and leaf number; and (iii) the content of antioxidants and phenols. We chose these crop species, due to their increasing widespread consumption and their nutritional properties [21]. Moreover, diet models, including row green leafy vegetables, have been recently associated with a low risk of severe pathologies, such as type II diabetes and cardiovascular diseases [22].

## 2. Results

### 2.1. Seed Germination

**Figure 1:**
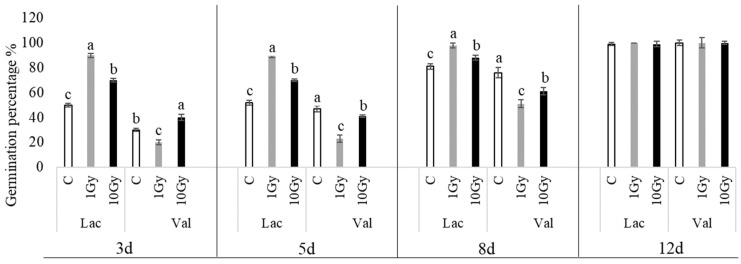
The percentages of seed germination in *L*. *sativa* (Lac) and *V*. *locusta* (Val) for control (C, white), 1 Gy (light grey), and 10 Gy (black) IR treatments. The bar charts represent the average percentages of seed germination, and the error bars represent the standard deviations. Different letters indicate significant differences according to Tukey’s test (*p* < 0.05).

The percentages of seed germination (Figure 1) indicate that both species reach 100% seed germination within 12 days. Specifically, in lettuce, treatment 1, shown in gray (Gy), yields a germination percentage of approximately 90 ± 3% after 3 days, and 100% after only 5 days, whereas the 10 Gy treatment shows a germination percentage significantly higher than the control, reaching 100% germination later (12 days). In corn salad, its germination was intrinsically slower than that for lettuce, even in control seeds; both treatments significantly decreased germination percentages compared to the control, but a recovery occurred between 8 and 12 d, an endpoint at which all of them are comparable to each other.

**Figure 2:**
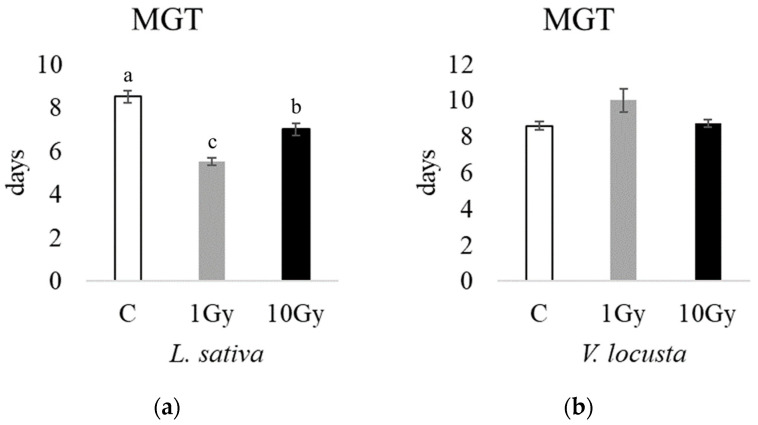
The mean germination times (MGTs) of *L*. *sativa* (**a**) and *V*. *locusta* (**b**) for control (C, white), 1 Gy (light grey), and 10 Gy (black) IR treatments. The bar charts represent the average MGTs, and the error bars the standard deviations. Different letters indicate significant differences according to Tukey’s test (*p* < 0.05).

Accordingly, the mean germination times (MGTs, Figure 2) were significantly lower in lettuce seeds at 1 Gy (5.5 days) and 10 Gy (7 days) compared to the control (8.5 days); however, the lamb’s lettuce MGT was not significantly different between the control and irradiated seeds.

### 2.2. Morphological Traits

**Figure 3:**
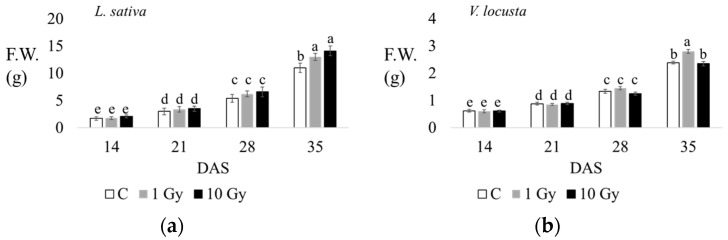
The fresh weight of aerial parts (g) of *L*. *sativa* (**a**) and *V*. *locusta* (**b**) for control (C, white), 1 Gy (grey), and 10 Gy (black) IR treatments at 14, 21, 28, and 35 days after sowing (DAS). The bar charts and each point of the trend time sub-figure show the average values, and the error bars represent the standard deviations. Different letters indicate significant differences according to Tukey’s test (*p* < 0.05).

In lettuce, the fresh weight (F.W.) of the aerial part was about 2 g after 14 d of growth, both in the control and treated seedlings; at 21 d, seedlings weight was about 3–4 g, without significant difference between the control and treated plants. At 28 d, plants from irradiated seeds showed a significantly higher weight. At 35 d, the FW was significantly higher in plants from irradiated seeds (Table 1, Figure 3 and Figure S2).

The fresh weights of the seedlings (aerial part) measured at each endpoint highlight significant increases at 1 and 10 Gy in lettuce, and at 1 Gy in corn salad at 35 days of culturing (Figure 3). The additional morphological traits measured at 35 d (Table 1), evidence that radiations promoted the growth of lettuce, increasing leaf number, root length, plant height, and both dry and fresh weights, with significant differences under the highest dose. However, in lamb’s lettuce, the stimulatory effect of radiations on plant growth reflected only on plant height and fresh weight, with significant differences at the 1 Gy dose (Table 1).

### 2.3. Total Antioxidant and Phenol Contents

**Figure 4:**
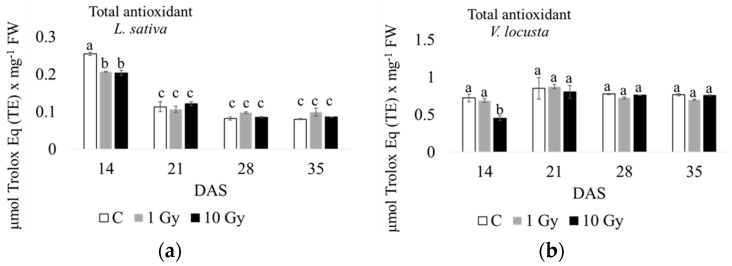
Total antioxidant content measured at 14, 21, 28, and 35 days after sowing (DAS) in *L*. *sativa* (**a**) and *V*. *locusta* (**b**) for control (C), 1 Gy, and 10 Gy IR treatments. The bar charts and each point of the trend time sub-figure represent the average values, and the error bars represent the standard deviations. Different letters indicate significant differences according to Tukey’s test (*p* < 0.05).

As for total antioxidants (Figure 4 and Figure S2), their content was 4–10-fold higher in corn salad than in lettuce. Moreover, in control plants of the latter, the highest level of antioxidants was measured at 14 days of culture compared to the other endpoints, decreasing during the growth period, with a concentration that was roughly constant at 28–35 days. Additionally, both the 1 Gy and 10 Gy treatments had significantly lower antioxidant concentrations at 14 days compared to the control, but this difference was recovered during plant growth. In the lamb’s lettuce control plants, an opposite trend was observed, with a slightly lower level of antioxidants at 14 d, increasing with a maximum concentration at 21 days, and maintaining a constant level at 28 and 35 days. In this species, only the 10 Gy treatment induced a significant decrease in antioxidants at 14 days, which recovered during the following culture weeks. In brief, in both species, radiation treatments seemed to decrease antioxidant concentrations at 14 days compared to the control, with a recovery during plant growth.

**Figure 5:**
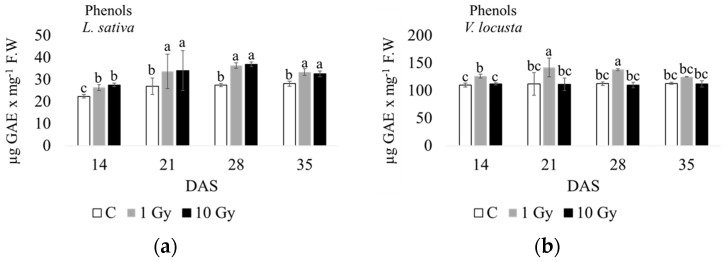
Phenol content measured at 14, 21, 28, and 35 days after sowing (DAS) in *L*. *sativa* (**a**) and *V*. *locusta* (**b**) for control (C), 1 Gy, and 10 Gy IR treatments. The bar charts and each point of the trend time sub-figure represent the average values, and the error bars represent the standard deviations. Different letters indicate significant differences according to Tukey’s test (*p* < 0.05).

In terms of phenolic compounds (Figure 5 and Figure S2), radiation stress significantly increased the content of phenols at each endpoint. In lettuce, this result was observed with both the 1 Gy and 10 Gy treatments, and a maximum was noted at 28 days. In corn salad, only 1 Gy enhanced phenol production, with a maximum occurring at 21 and 28 days, with the phenol content in plants from 10 Gy seeds not significantly different from the control. 

## 3. Discussion

The present research highlights that low-dose X-rays boosted growth in both of the studied lettuce species by increasing biomass production, without altering the ratio between dry and fresh weight. In lettuce under radiation treatment, the increase in weight affected all morphological parameters measured at 35 d of culturing; in corn salad, only plant height and fresh weight were affected. Overall, the results suggest that seed treatment with high-energy radiation could be a valuable option to increase the yields of ready-to-eat crop species. These findings are noticeable considering that *L*. *sativa* and *V*. *locusta* are among the most economically important salad crop species; in fact, their consumption has spread remarkably due to their beneficial nutritional properties and phytochemical contents [3,23,24]. 

Species-specific behavior under the different doses was observed in the two species; in *L*. *sativa*, both doses increased biomass production in contrast to *V*. *locusta*, whose biomass significantly increased only under the lower dose. This indicates that the use of radiations in seed priming needs to be customized to the species. Accordingly, literature reports focused on seed irradiation by X-rays show that a stimulatory effect was observed at a dose lower than 1 Gy in *Phoenix dactylifera* [25], and at 10 Gy in *Solanum lycopersicon* [26]. A further difference between the species under study was the effect of priming on seed germination. In general, seed priming decreases the MGT because it interrupts dormancy, activates protein synthesis at the base of the main metabolic pathways, and mobilizes reserve substances, bringing the seed to a state that immediately precedes the breaking of the integuments and the emergence of the roots; this sequence of events is triggered by priming-induced stress [7]. This condition occurred only in *L*. *sativa*, since in *V*. *locusta* the MGT of irradiated seeds was not different from the control. 

Nonetheless, the significant low level of antioxidants observed in 14-day-old plants, for both species from irradiated seeds could be interpreted as an early sign of stress induced by radiation, a stress that was promptly recovered from since antioxidant concentrations reached levels comparable to the control in one week. Apart from the low level of antioxidants measured at the early growth phase in plants from irradiated seeds, our results showed that in general, antioxidant concentrations were not different in treated plants compared to controls in both salad species. This result is not in line with most published papers. Indeed, a large body of literature highlights the relationships between abiotic stress, oxidative damage with ROS production, and an increase in enzymatic and/or non-enzymatic antioxidants to counteract stress (e.g., [27]). However, recent literature also highlighted a positive role of ROS during seed germination in terms of signaling molecules breaking seed dormancy and providing protection against pathogens [28]. Therefore, the low level of antioxidants observed in the plants could be due to the fact that low-dose X-ray radiation probably resulted in moderate ROS production in the dry seeds, thereby stimulating germination and subsequent plant growth, rather than causing stress conditions. Of course, we cannot exclude that an increase in total antioxidants could occur in a different time frame from those considered in this research, or under different doses of radiation. In any case, antioxidant concentrations reported for lettuce and corn salad in control conditions are in line with our findings; other authors found a total antioxidant content that is higher in *V*. *locusta* compared to *L*. *sativa* [29,30,31,32]. However, a proper comparison is not practicable due to the different detection protocols and plant ages. The results regarding total antioxidants measured in the two species deserve some additional considerations. Firstly, the decreasing trend of antioxidant concentrations found in lettuce during plant growth may be explained in terms of dilution effect [33,34] due to leaf growth by cell distention, which was especially evident between 14 and 21 days (Figure 4a and Figure S2). Secondly, the substantially similar concentrations of antioxidants in controls and plants from irradiated seeds, in contrast with previous literature data, could be compensated by the simultaneous increase in phenols. Some authors [29], found a linear relation between antioxidants and phenolic compounds in *Lactuca sativa*; instead, our experiment highlighted in both species a significant increase in phenols during the growth of plants from irradiated seeds compared to controls. It has been reported that most phenols contribute to plant growth, specifically in cell wall formation; in fact, phenols as hydroxycinnamic acids were found in the insoluble or cell wall fraction as esters [35]. These pools of wall-bound phenolic acids act as a reservoir of phenylpropanoid units for lignin biosynthesis, or they may even represent the beginnings of lignification itself. Moreover, these esters, with a wide population of derived molecules, also have a regulatory role, being responsible for transduction of light energy leading to a relaxation of the cell wall in conjunction with water flux into the cell, increasing turgor pressure, and growth, again supporting the occurrence of a dilution effect [30,31]. Phenolic compounds counteract nutrition stress, cold stress, and radiation, thus providing resistance to the plant [35]; however, literature reports specifically refer to UVB radiation [36] inducing phenol production in plant leaves, as a screen of high-energy solar radiation. Although no data have been reported for X-rays, it could be hypothesized that a similar behavior may occur in plants under this stress condition, since X-rays are an ionizing radiation as well.

Finally, since plants are sessile organisms, they cannot escape from stress; therefore, they have developed peculiar adaptive responses. Specifically, under low stress conditions, faster growth can sometimes occur, allowing plants to overcome critical phases such as seed germination, in which the organism is more fragile [37]. According to these considerations and our results, we hypothesize that low-dose X-ray radiation could probably induce moderate ROS production in dry seeds, thereby stimulating germination and subsequent plant growth. 

## 4. Materials and Methods

### 4.1. Plant Material

*Lactuca sativa* L. var. longifolia (romain lettuce; Asteraceae) and *Valerianella locusta* L. Laterr. (cultivar verte de cambrai; Valerianaceae) are two edible species commonly used as salads. Being crop species, they offer the advantage of having fundamentally uniform characteristics, which guarantees a product that does not vary over time, which in turn facilitates the evaluation of differences in morphophysiological traits occurring under each experimental condition. Furthermore, based on literature reports [38,39,40,41], valerian and lettuce have an intrinsically different production of antioxidants and phenols, and since these metabolites are regarded as good indicators of the activation of strategies to counteract stress, different responses may occur in the two species.

### 4.2. Seed Irradiation 

Dry seeds of *Lactuca sativa* and *Valerianella locusta* were irradiated with 1 and 10 Gy X-rays at the Radiation Biophysics Laboratory (Department of Physics, University of Napoli Federico II) by means of a radiogenic tube (STABILIPAN II; Siemens, Berlin, Germany) following the method of [38]. Doses were administered through a 1-millimeter Cu filtration at a dose rate of about 1.36 Gy min^−1^. Dosimetry was routinely checked using an ionization chamber to ensure dose uniformity within a square field of 225 cm^2^. 

### 4.3. Seed Germination

Irradiated seeds and non-irradiated seeds used as control were germinated in the dark at 25 °C on wet filter paper (5 mL water in 9 cm diameter Petri dishes containing two paper discs). The seed germination percentages were evaluated using 50 seeds per species and treatments at 3, 5, 8, and 12 days. Since different batches of seeds can have the same final percentage of germination yet differ in the speed or uniformity of sprouting, we also calculated the mean germination time (MGT), which provides more precise information on the speed and uniformity of germination. The MGT was calculated according to the formula reported in [42,43]:

MGT = Σ(ni × ti)/N

where ti is the time from the start of incubation in water; ni is the number of seeds completing germination at time ti; N is the total number of germinated seeds at the end of the test. Seeds with a 1 mm long radicle were considered germinated [44]. Radicle length was measured under a stereomicroscope at ×10 magnification. Culture set up and observations.

Forty seedlings with fully developed cotyledons for each thesis were transplanted to a hydroponic system at pH 5.8 in Hoagland solution, as described in scientific papers [45,46]. The Hoagland solution required specific macro- and micronutrients, reported as follows: 2M·KNO_3_, (NO_3_)_2_·4H_2_O, 2M·MgSO_4_·7H_2_O, 2M·NH_4_H_2_PO_4_, and 1M·KH_2_PO_4_ as macronutrients; H_3_BO_3_, MnCl_2_·4H_2_O, ZnSO_4_·7H_2_O, CuSO_4_·5H_2_O, and H_2_MoO_4_·H_2_O as micronutrients; and chelated iron prepared by dissolving 6 gr of Na_2_EDTA and 7 gr of Fe(NH4)_2_(SO4)_2_·6H_2_O in 1L of distilled water and adjusting the pH to 7.5. The final solution of chelated iron was used by adding 5 mL per liter of Hoagland medium. The growth chamber was kept at the following conditions: (1) temperature 25/18 °C; (2) relative humidity (RH) 60–75% (day/night); (3) photoperiod of 16 h of light per day with a photosynthetic photon flux density (PPFD) at the top of the canopy of 180–190 μmol photons m^−2^ s^−1^.

Morpho-physiological traits (i.e., fresh weight of the plant, total antioxidant content, and total phenol content) were measured in 10 plants per species and treatments at 14, 21, 28, and 35 days of culturing. At 35 days, the culture dry weight (after drying at 40 °C in an oven until constant weight), plant height, leaf number, and root length were also measured. Specifically, we measured the plant height from the collar to the end of the highest leaf (Figure S1); the root length was measured from the collar to the longest tip of the primary root.

### 4.4. Antioxidant Content

The antioxidant analysis was carried out using the ferric reducing antioxidant power assay (FRAP) according to [47]. Homogenized fresh leaf samples, 250 mg each, were resuspended with 60:40 (*v*/*v*) methanol/water solution and centrifuged at 14,000 rpm for 15 min at 4 °C. Then, acetate buffer containing 10 mM tripyridyltriazine (TPTZ) in 40 mM HCl, (1:1.6) and 12 mM·FeCl_3_ (1:16) was added to the extract (1:16 300 mM pH 3.6); After 1 h of incubation at 4 °C, the absorbance was measured at 593 nm with a spectrophotometer (UV-VIS Cary 100; Agilent Technologies, Palo Alto, CA, USA) using Trolox (6-hydroxy-2,5,7,8-tetramethylchroman-2-carboxylic acid) as standard. The total antioxidant capacity was expressed as μmol Trolox equivalents per mg of fresh sample.

### 4.5. Total Phenolic Compounds (TPC)

Folin–Ciocalteu (F–C) reagent was used to quantify the total phenolic compounds (TPC) [48]. The F–C assay was carried out according to [38]. This test is based on the oxidation of phenols in alkaline solution, with the transfer of electrons to phosphomolybdic/phosphotungstic acid complexes, whose reduced form appears blue. The phenolic concentration was determined via spectroscopy by its absorbance at 760 nm. Although the redox reaction is not specific for phenolic compounds, the extraction procedure eliminates more than 85% of potentially interfering compounds [49]. A mass of 250 mg of ground homogenized fresh tissue was resuspended in 1 mL of 60% methanol and stored on ice for 3 min in the dark; the volume was brought to 5 mL with the addition of 60% methanol. Then, the samples were centrifuged at 13,000 rpm for 5 min. Then, 62.5 μL of supernatant were added to an equal volume of Folin–Ciocalteu’s reagent (Sigma, St. Louis, MI, USA) and 250 μL of deionized water and incubated for 6 min at room temperature. Finally, the addition of 625 μL of 7.5% sodium carbonate and 500 μL of deionized water followed by incubation for 90 min at room temperature produced an alkaline environment that was necessary for the redox reaction. The absorbance was measured at 760 nm. Total phenols were expressed in μg of gallic acid equivalents/mg of fresh weight, after drawing a calibration curve using gallic acid as a standard in a range of 0–125 μg.

### 4.6. Data Analyses

The data were analyzed with one- or two-way ANOVA, using IBM SPSS Statistics for Windows (IBM Corp. Released 2020, Version 27.0; IBM Corp, Armonk, NY, USA). The normality and homogeneity of the variance of the data set were evaluated using the Shapiro–Wilk and Levene tests, respectively. As for the percentage data, the analysis was performed after arcsine transformation. Multiple comparison tests were performed with Tukey’s post hoc test (*p <* 0.05).

## 5. Conclusions

The present study highlights that, in comparison with control plants from non-irradiated seeds, small doses of radiation administered to the seeds of *L*. *sativa* induced a priming effect, since the treatments significantly decreased the mean germination time (MGT), with a subsequent boosting effect on growth and increase in phenol content. In contrast, the MGT did not increase significantly in *V*. *locusta*, in which a boosting of the growth and an increase in phenol content occurred only at 1 Gy compared to control plants. These findings are notable, considering that *L*. *sativa* and *V*. *locusta* are among the most economically important salad crop species, with increasing consumption due to their beneficial nutritional properties. Overall, the results suggest that seed treatment using high-energy radiation could be a valuable option to increase the yields of ready-to-eat crop species. However, the species-specific behaviors observed in the two species indicate that the use of radiations in seed priming needs to be customized to the species.

## Figures and Tables

**Table 1:** Morphological traits at 35 days (mean ± standard deviation, *n* = 10).

*L. sativa*						
	Leaf Number	Root Length (cm)	Plant Height (cm)	F.W. Aerial Part (g)	D.W. Aerial Part (g)	D.W./F.W.
**C**	10.8 ± 1.2 **b**	21.1 ± 1.9 **b**	17.8 ± 1.2 **b**	11 ± 0.85 **c**	0.6 ± 0.1 **b**	5%
**1 Gy**	10.8 ± 0.9 **b**	28.6 ± 4.3 **ab**	18.1 ± 1.4 **ab**	13 ± 0.65 **b**	0.7 ± 0.1 **a**	5%
**10 Gy**	12.1 ± 1.2 **a**	35.5 ± 10.8 **a**	19.3 ± 0.9 **a**	14.1 ± 0.9 **a**	0.7 ± 0.1 **a**	5%
*V*. *locusta*						
**C**	7.7 ± 1.2	13.8 ± 1.3	4 ± 0.7 **b**	2.4 ± 0.1 **b**	0.1 ± 0.05	4%
**1 Gy**	8.6 ± 0.5	14.9 ± 2.7	5.2 ± 0.5 **a**	2.8 ± 0.1 **a**	0.1 ± 0.07	4%
**10 Gy**	8.3 ± 0.9	13.2 ± 1.2	4.2 ± 0.4 **b**	2.3 ± 0.1 **b**	0.1 ± 0.05	4%

Different letters indicate significant differences according to Tukey’s test (*p <* 0.05).

## Data Availability

All data are reported in the text and in the Supplementary Materials.

## Supplementary Materials

The following supporting information can be downloaded at: https://www.mdpi.com/article/10.3390/plants13020165/s1, Figure S1. Twenty-one-day-old seedlings. (A) *L*. *sativa*; (B) *V*. *locusta*. Figure S2. Results of the two-way ANOVA and time trends with reference to the Figures 3 (a,b), 4 (c,d) and 5 (e,f) of the text. In red, factors significantly affecting the dependent variables.
