# Supplementary material for: Seed Priming by Low-Dose Radiation Improves Growth of Lactuca sativa and Valerianella locusta
Source: Plants (Basel). 2024 Jan 8;13(2):165. doi: 10.3390/plants13020165 (PMC10818939; doi:10.3390/plants13020165)
Supplement: Supplementary file 1 [file plants-13-00165-s001.zip › Figure S2.pdf]

L.sativa

2-way ANOVA	DoF	SS	MS	F	p
**Treatment**	2	11	5.6	16	0.000
**Time**	3	665	222	645	0.000
**Treatment*Time**	6	8.6	1.4	4.2	0.005
**Error**	24	8.25	0.344		
**Total**	35	693			

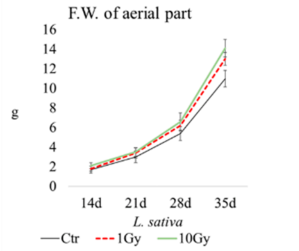

a)

V. locusta

2-way ANOVA	DoF	SS	MS	F	p
**Treatment**	2	0.14	0.07	26	0.000
**Time**	3	1895161	631720	2,301	0.000
**Treatment*Time**	6	0.26	0.04	16	0.000
**Error**	24	0.07	0.002		
**Total**	35	1941750			

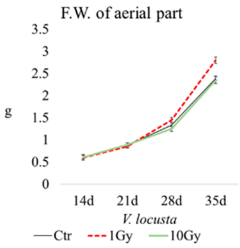

b)

2-way ANOVA	DoF	SS	MS	F	p
**Treatment**	2	0.00041	0.00021	5	0.014
**Time**	3	0.1091	0.03637	905	0.000
**Treatment*Time**	6	0.00576	0.00096	23.89	0.000
**Error**	24	0.00096	0.00004		
**Total**	35	0.11624			

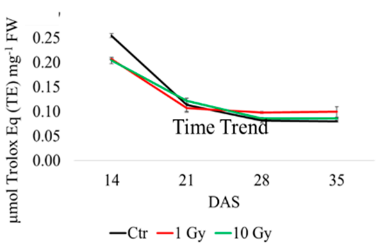

c)

2-way ANOVA	DoF	SS	MS	F	p
**Treatment**	2	0.04332	0	8	0.003
**Time**	3	0.22426	0	26	0.000
**Treatment*Time**	6	0.10455	0	6	0.001
**Error**	24	0.068	0.00283		
**Total**	35	0.44013			

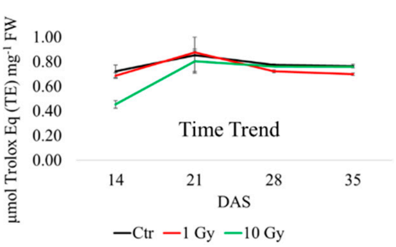

d)

2-way ANOVA	DoF	SS	MS	F	p
**Treatment**	2	760	380	27	0.000
**Time**	3	776	258	18	0.000
**Treatment*Time**	6	71	12	0.9	0.500
**Error**	72	1017	14		
**Total**	83	2627			

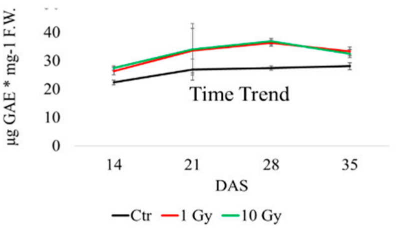

e)

2-way ANOVA	DoF	SS	MS	F	p
**Treatment**	2	10854	5427	69	0.000
**Time**	3	620	207	3	0.050
**Treatment*Time**	6	1380	230	2.93	0.010
**Error**	96	7541	79		
**Total**	107	20395			

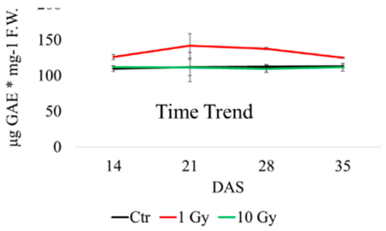

f)

Figure S2. Results of the two-way ANOVA and time trends with reference to the Figures 3 (a,b),4 (c,d) and 5 (e,f) of the text. In red, factors significantly affecting the dependent variables.
