# Supplementary figures and images for: Seed Priming by Low-Dose Radiation Improves Growth of Lactuca sativa and Valerianella locusta
Source: Plants (Basel). 2024 Jan 8;13(2):165. doi: 10.3390/plants13020165 (PMC10818939; doi:10.3390/plants13020165)

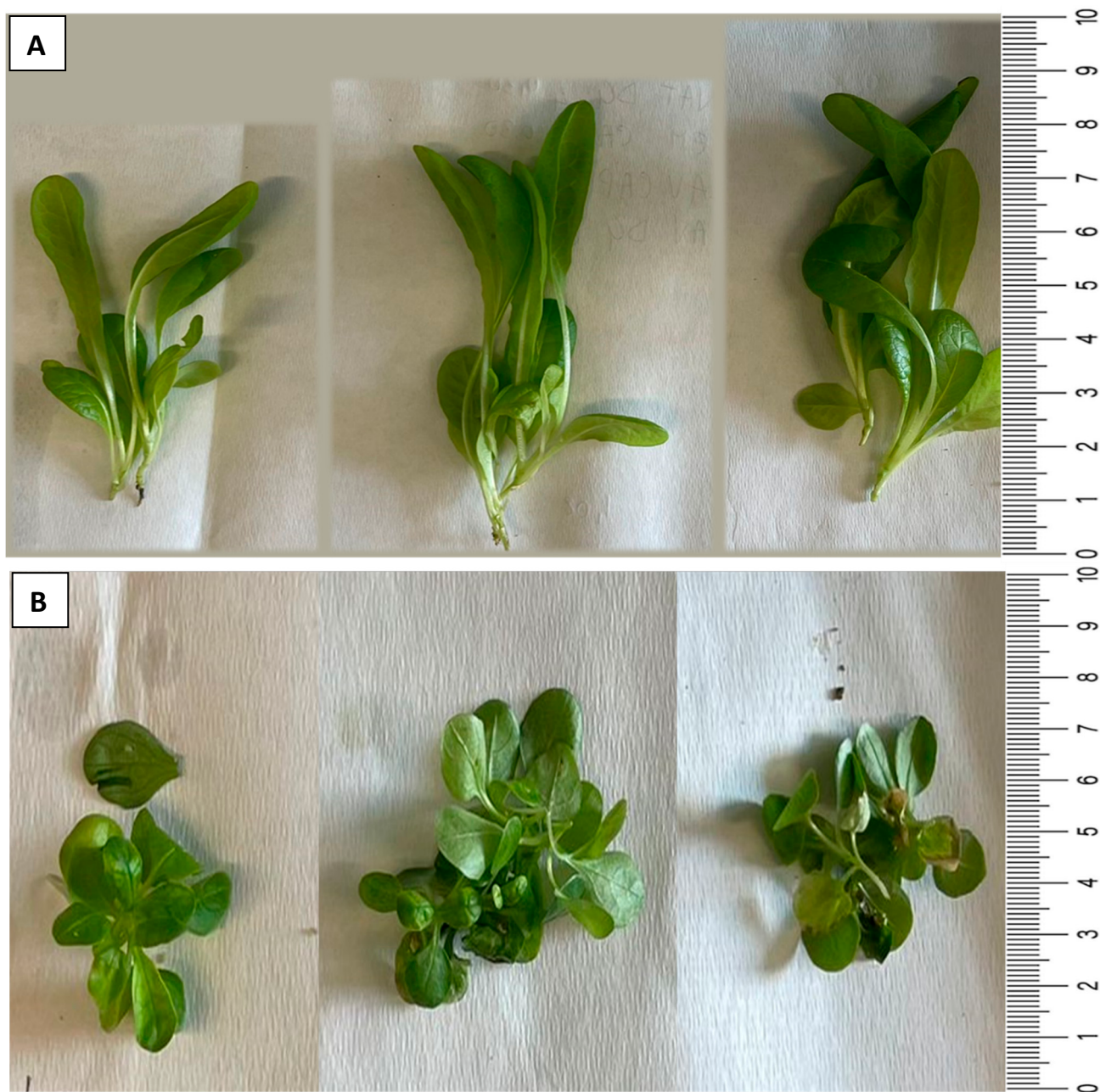

**Figure S1.** 21 day old seedlings. A) *L. sativa*; B) *V. locusta*

Supplement: Supplementary file 1 [file plants-13-00165-s001.zip › Figure S1.pdf]
